# Evaluating dried pomegranate peel as a functional feed additive: effects on growth, carcass traits, and gut health in broilers

**DOI:** 10.1007/s11250-025-04455-y

**Published:** 2025-05-15

**Authors:** Mohammed Younis, Saber G. Abdo, Mohammed A. Abu Elmakarem, Fatma El-Zahraa A. Mustafa, Mohamed A. Fawaz

**Affiliations:** 1 Department of Animal Production, Faculty of Agriculture, Al-Azhar University, Assiut, 71524 Egypt; 2 Department of Cell and Tissues, Faculty of Veterinary Medicine, Assiut University, Assiut, 71515 Egypt; 3 Department of Animal and Poultry Production, Faculty of Agriculture, South Valley University, Qena, 83523 Egypt

**Keywords:** Pomegranate peel, Feed additives, Broiler chicks, Growth performance, Health status

## Abstract

This study investigated the impact of dietary supplementation with dried pomegranate peel (DPP) on growth performance, carcass attributes, blood parameters, and cecal microbiota of broiler chickens. A total of 120 one-day-old Ross 308 broiler chicks were randomly assigned to three experimental groups: a control group (basal diet without any supplementation), and two treatment groups with diets supplemented with 1% and 2% DPP, respectively. Growth performance was monitored weekly, and carcass attributes were measured at the end of the 42-days trial. Blood samples were collected for biochemical analysis, and cecal microbiota (total bacterial count, *E. coli* and *salmonella*) was assessed. Results indicated that DPP supplementation significantly reduced body weight, body weight gain, and feed consumption compared to the control group, particularly at 21 and 42 days. Birds fed 2% DPP showed a better feed conversion ratio (FCR) but body weight was reduced during both the starter period and overall experimental period, while those fed 1% DPP had an improved FCR only during the starter phase but showed a worsened FCR during the finisher and overall period. DPP supplementation also significantly increased the relative weights of the eviscerated carcass, liver, heart, gizzard, and spleen while reducing abdominal fat. Blood analysis revealed no significant effects on protein or liver enzymes, but DPP reduced glucose, cholesterol, HDL, and triglyceride levels. Additionally, DPP reduced the cecal counts of total bacteria, *E. coli*, and *Salmonella*. Histological analysis revealed that DPP supplementation improved bursal and ileal structures, enhancing immunity and gut health. In conclusion, DPP supplementation, particularly at 2%, improved gut health, reduced abdominal fat, and modulated microbial populations but adversely affected growth performance due to reduced feed palatability. These findings suggest that DPP may be a functional feed additive with health-promoting benefits, though further research is needed to optimize its inclusion level.

## Introduction

The poultry industry is one of the most important and fastest-growing sectors of agriculture production worldwide (Mottet and Tempio 2017; Erdaw and Beyene 2022). Additionally, poultry products, such as eggs and meat, serve as a cost-effective and easily digestible source of animal protein (Wahyono and Utami 2018; Nkukwana 2018). However, the poultry industry faces numerous challenges, such as increased feeding costs, which represent about 60–70% of total production cost (Thirumalaisamy et al. 2016), and limited availability of feedstuffs amid rising prices (Hafez and Attia 2020). Additionally, growing concerns over antibiotic-resistant bacteria have prompted many countries to prohibit the use of antibiotic-growth promoters (Salim et al. 2018). These challenges, along with the global approach to sustainable development in poultry production, lead nutritionists’ attitudes to look for natural alternatives to antibiotic growth promoters (Bist et al. 2024). In poultry production, plant-based feed additives have become the most widely used natural alternative to antibiotic growth promoters (Gadde et al. 2017). Due to their strong antibacterial, antiviral and antioxidant properties, (Parham et al. 2020). One such promising alternative is the use of pomegranate peel powder (PPP) as a feed additive in broiler diets.

Pomegranate (*Punica granatum* L.), a fruit known for its rich antioxidant properties, has gained significant attention in recent years for its potential health benefits (Fourati et al. 2020; Fahmy and Farag 2022; Valero-Mendoza et al. 2023). About 30 to 40 percent of the pomegranate fruit is wasted in the form of peel, as a by-product of the pomegranate juice industry (Singh et al. 2019). Notably, pomegranate peels are rich in bioactive compounds, including phenolics, flavonoids, tannins, vitamin C and vitamin E (Magangana et al. 2020; Marra et al. 2022). Important minerals including calcium, potassium, phosphorus, calcium, manganese, sodium, iron and nitrogen are also found in pomegranates (Akuru et al. 2020). Elzoghbiy et al. (2022) reported that the quantitative analysis of pomegranate peel extract revealed the presence of total flavonoids (304.21 ± 0.43 mg/g rutin), total phenolic acids (352.60 ± 0.54 mg/g gallic acid), total tannins (1.66 ± 0.11%), total saponins (2.67 ± 0.13%), and total alkaloids (1.51 ± 0.17%). These compounds are associated with various health benefits, including antimicrobial, anti-inflammatory, antioxidant, anticancer, and anti-cardiovascular properties (Siddiqui et al. 2024). In addition, the peel of the pomegranate contains a significant source of organic acids, including fumaric, citric, malic, acetic, oxalic, tartaric, lactic, and ascorbic acids, as well as numerous other nutrients (Poyrazoğlu et al. 2002). By acidifying the digestive tract and lowering the pH level in the surrounding environment, the organic acids may increase the chicken’s resistance to infections and make it more difficult for certain intestinal pathogens, such E. coli, to proliferate (Khan et al. 2022). According to studies, the amount of E. coli was considerably lower in the 4% pomegranate peel powder group than in the control group (*P* < 0.05) (Ghasemi-Sadabadi et al. 2021). Because of their superior nutritional and chemical makeup, pomegranate peels are used in a wide range of dishes on a daily basis (Kandylis and Kokkinomagoulos 2020). Pomegranate peel’s chemical formula indicated that it comprises (4.90–8.97%) crude protein, (3.40–4.22%) ash, (16.30%–19.41%) crude fiber, (0.85%–1.26%) ether extract, and (59.60%) carbs (El-Hamamsy and El-khamissi 2020). This makes them suitable for use as a natural food additive (Gullón et al. 2020). The disposal of pomegranate peel waste poses environmental challenges due to its massive production (Ain et al. 2023).

Several studies have explored the effects of natural feed additives on the growth performance, immune response, and meat quality of broiler chickens. For instance, Elnaggar et al. (2022) stated that adding 0.25, 0.50, 1.0, or 1.5% of pomegranate peel powder to broiler diet significantly increased body weight and body weight gain, improved the feed conversion ratio, and reduced the percentage of the abdominal fat compared to the control group. Gosai et al. (2023) found that adding 1% pomegranate peel to the broiler diet significantly improved final body weight and body weight gain, improved the feed conversion ratio, and increased the return over feed cost compared to the control group.

However, despite these promising findings, research on the optimal inclusion levels and long-term effects of pomegranate peel powder in broiler diets remains limited. This necessitates further investigation to fully understand the potential benefits and any possible drawbacks of incorporating PPP into poultry feed. This study investigates the potential of pomegranate peel powder as a dietary supplement in broiler chick diets. It evaluates its impact on growth performance, feed efficiency, immune response, meat quality, oxidative stress markers, and overall health. The research aims to explore the feasibility of using pomegranate peel as a sustainable feed additive in the poultry industry. By incorporating pomegranate peel, the study aligns with circular economy principles, reducing waste, lowering feed costs, and promoting agricultural sustainability.

## Materials and methods

### Birds and housing

This study was conducted on the poultry farm, department of Animal Production Faculty of Agriculture et al.-Azhar University, Assiut, Egypt, where a tropical environment predominates, the experiment adhered to protocols approved by the Animal Health and Care Committee and the ethical committee of the Faculty of Veterinary Medicine, Assiut University, Egypt, following OIE standards (Approval No. 06/2025/0300). A total of 120 one-day-old Ross 308 broiler chicks were used in this study. The chicks were randomly allocated to three experimental groups, with each group consisting of 40 birds, distributed across four replicates of 10 chicks. The chicks were housed in floor pens under standard environmental conditions according to the Ross 308 management guidelines. Temperature, lighting, and ventilation were maintained according to commercial standards for broiler rearing. Feed and water were provided *ad libitum* throughout the 42-day experimental period.

### Experimental design and diets

The chicks were fed isocaloric and isonitrogenous diets, with the only difference being the inclusion of dried pomegranate peel (DPP) at varying levels. The experiment consisted of the following treatment groups:

1. Control Group: Basal diet with no pomegranate peel supplementation.
2. DPP 1% Group: Basal diet supplemented with 1% dried pomegranate peel.
3. DPP 2% Group: Basal diet supplemented with 2% dried pomegranate peel.

The diets were formulated to meet or exceed the nutrient requirements of Ross 308 broiler chickens, based on the recommendations of the National Research Council (NRC 1994). The dried pomegranate peel was prepared following the method described by Mphahlele et al. (2016), where the peels were oven-dried at 60 °C for 24 h to preserve bioactive compounds and minimize degradation. After drying, the peels were finely ground using a mechanical grinder and stored in airtight, light-protected containers at room temperature to prevent oxidation and maintain stability. Moisture content was carefully controlled before storage to prevent microbial growth and the deterioration of active compounds. The formulation and the chemical composition of the starter and finisher are presented in Table 1.

**Table 1:** composition and calculated analysis of experimental diets

Ingredients	Ingredients percentages	
	Starter	Grower
Yellow corn	56.5	60.1
Soybean meal (C*P-*44%)	30	23.4
Gluten (C*P-*60%)	7.54	8.54
Vegetable oil (mixture)	1.8	3.9
Methionine	0.06	0.01
Lysine	0.1	0.1
Limestone	1	1
Dicalcium phosphate	2.35	2.30
Salt (NaCl)	0.35	0.35
Premix ^*^	0.3	0.3
Total	100	100
Calculated analysis		
ME Kcal/kg diet	3000	3200
Crude protein	23	21
Crude fat	4.38	6.58
Crude fiber	3.53	3.14
Methionine	0.52	0.45
Lysine	1.19	1.02
Calcium	1.1	1
Available phosphorus	0.52	0.50

^*^ each kg premix contained: vita. A (acetate), 6,250,000 I.U.; vita D3 (Cholecalciferol), 2,500,000 I.U.; vitamin E (α – tocopherol), 25,000 mg; vitamin k,1750 mg; vitamin B1, 500 mg; vitamin B2, 2750 mg; vitamin B6, 1250 mg; vitamin B12, 10 mg; nicotinic acid (niacin), 20000 mg; Calcium pantothenate, 5000 mg; folic acid, 500 mg; biotin 50 mg; iron sulfate,22,000 mg; manganese oxide,31,000 mg; copper sulfate,2500 mg; zinc oxide,37,500 mg; potassium iodide,650 mg; sodium selenite, 113 mg; cobaltous sulfate,50 mg; Ethoxyquin,250 mg; wheat bran (carrier), 120 gm; limestone (carrier), up to 1 kg

### Growth performance measurement

Body weight and feed consumption were recorded weekly. From these data, the cumulative body weight gain, cumulative feed consumption, and feed conversion ratio (FCR) were calculated at 21 days old and at the end of the experiment on day 42. Mortality was recorded as it occurred, and dead birds were weighed to adjust FCR calculations.

### Carcass traits measurement

At the end of the 42-day trial, two birds per replicate (a total of eight birds per group) with body weight close to the average body weight of their replicate were selected and slaughtered following standard ethical procedures. The birds were eviscerated, and the carcass traits including absolute and relative weights of the eviscerated carcass, dressed carcass, internal organs (liver, heart, gizzard, spleen,) and abdominal fat were measured.

### Blood collection and analysis

Blood samples were collected from the jugular veins of the selected birds at the end of the experiment. The blood was placed in plain tubes to allow clotting, after which serum was separated by centrifugation at 4000 RPM for 15 min. The serum biochemical parameters (total protein, albumin, glucose, triglycerides, cholesterol, HDL, LDL, ALT, AST, TAC, and MDA) were analyzed using commercial reagent kits from Egyptian Co for Biotechnology—Spectrum Diagnostics (S.A.E) and a UV/VIS Spectrophotometer (Model: Optizen 3220UV None-PC, Korea). The assessment of blood parameters was conducted according to the instructions provided by the reagent kits manufacturer. Globulin was calculated as the difference between the concentration of total protein and albumin.

### Cecal microbial analysis

At the time of slaughter, samples of the cecal content were aseptically collected from each selected bird for microbial analysis. Approximately 1 g of cecal digesta was collected, homogenized, and serial dilutions were prepared for the enumeration of total bacteria, Escherichia coli (E. coli), and Salmonella. The microbial populations were determined using selective agar media. Plates were incubated at 37 °C for 48 h, and colony-forming units (CFUs) were counted.

### Histomorphometry of bursa and ileum

Tissue samples of the bursa of Fabricius and ileum were collected from experimental birds immediately after euthanasia. Tissue samples were gently rinsed with phosphate-buffered saline (PBS, pH 7.4) to remove residual contents and fixed in 10% neutral-buffered formalin for 48 h. Fixed tissues were dehydrated through a graded series of ethanol (70%, 80%, 90%, and 100%), cleared in xylene, and embedded in paraffin wax. Sections were cut to a thickness of 4–5 µm and stained with hematoxylin and eosin (H&E) for general histological examination. The histological slides were examined under a light microscope equipped with a digital camera. High-resolution images were captured, and morphometric parameters were measured using image analysis software (ImageJ).

### Statistical analysis

The obtained data were statistically analyzed using one-way analysis of variance (ANOVA) following the General Linear Model (GLM) procedures in SAS (2009). Duncan’s multiple range test was used to determine significant differences between treatment means. Results were expressed as means ± standard error, with statistical significance set at P ≤ 0.05.

Statistical Model: $${\text{Y}}_{\text{ij}}=\upmu +{\text{T}}_{\text{i}}+{\text{e}}_{\text{ij}}$$

Where: ${\text{Y}}_{\text{ij}}$ = observed dependent variable, µ = overall mean, T_i_ = effect of the ${i}^{th}$ treatment, ${\text{e}}_{\text{ij}}$= random error term.

## Results and discussion

### Growth performance

During the experimental periods, there was no mortality. Dietary dried pomegranate peel (DPP) supplementation effects on live body weight, body weight gain, feed consumption, and feed conversion ratio are presented in Table 2. Broiler chicks fed diets supplemented with DPP had significantly lower body weight and weight gain than the control group at 21 and 42 days. Also, birds in the treated groups consumed less feed during the starter phase and the entire trial. Regarding feed conversion ratio (FCR), chicks fed 2% DPP showed the best FCR during the starter phase and the entire trial, but during the finisher phase and overall period, those fed 1% DPP had the poorest FCR among all treatments. Pomegranate peels are a rich source of biologically active compounds, including phenols, flavonoids, tannins, vitamin C, and vitamin E (Magangana et al. 2020; Marra et al. 2022). These bioactive components exhibit a broad spectrum of health-promoting properties, such as antimicrobial, anti-inflammatory, antioxidant, anticancer, and anti-cardiovascular effects (Siddiqui et al. 2024). Pomegranate peels serve as a primary raw material for tannin production due to their high tannin content (3.2–3.8 mg catechin equivalent/g dry weight) and abundant availability (Saad et al. 2012). While tannins possess beneficial properties, including antimicrobial and antioxidant activities, they also exhibit astringent properties, which can reduce feed palatability and intake (Bee et al. 2016; Naumann et al. 2017). The negative effects of tannins on broiler performance have been documented. Hidayat et al. (2021) observed that high dietary tannin levels reduced average daily gain (ADG) and feed intake, likely due to the inhibitory effects on appetite and nutrient absorption. Al-Muammar and Khan (2012) noted that pomegranate extract can influence appetite regulation by decreasing leptin levels and increasing adiponectin levels, hormones associated with energy balance and fat metabolism. This hormonal modulation may explain the observed reduction in feed intake in broiler chickens fed pomegranate peel-supplemented diets. Consequently, the lower feed intake negatively impacted body weight and body weight gain in these groups.

**Table 2:** Effect of supplementing broiler diets with different levels of pomegranate peel on growth performance from 1 to 42 days of age

Variables	C	DPP1	DPP2	SEM	*P-*value
Live body weight (g)					
0- day	41.77	41.83	41.83	0.16	0.9819
21 days	961.90^a^	854.30^b^	829.60^c^	6.28	<.0001
42 days	2662.30^a^	2428.23^b^	2427.93^b^	13.11	<.0001
Body weight gain (g)					
0–21 days	920.13^a^	812.47^b^	787.77^c^	6.26	<.0001
21–42 days	1700.40^a^	1573.93^b^	1598.33^b^	8.49	<.0001
0–42 days	2620.53^a^	2386.40^b^	2386.10^b^	13.10	<.0001
Feed consumption					
0–21 days	1189.90^a^	1017.93^b^	961.10^c^	34.50	<.0001
21–42 days	2495.03	2410.80	2323.00	34.90	0.1203
0–42 days	3684.93^a^	3428.73^b^	3284.10^b^	64.00	0.0042
Feed conversion ratio					
0–21 days	1.29^a^	1.25^b^	1.22^c^	0.004	<.0001
21–42 days	1.47^b^	1.53^a^	1.45^b^	0.008	<.0001
0–42 days	1.41^b^	1.44^a^	1.38^c^	0.005	<.0001

^a,^^b,c^ Different superscript letters within a row indicate significant differences between means at a specified significance level (*P* < 0.05 or *P* < 0.01). C: Control (basal diets); DPP1; basal diets supplemented with dried pomegranate peel at 1%.; DPP1; basal diets supplemented with dried pomegranate peel at 2%

Our findings align with those of Ghasemi-Sadabadi et al. (2021), who observed significant reductions in BW, BWG, feed intake, and FCR at higher inclusion levels of pomegranate peel (8%) in broiler diets. However, contrasting results have been reported. Studies by Akuru et al. (2020), Kamel et al. (2021), and Elnaggar et al. (2022) showed improved growth performance and FCR with pomegranate peel powder supplementation. Moreover, recent investigations by Elbaz (2023) and Xu et al. (2024) indicated that incorporating pomegranate peel powder into broiler diets under heat stress or E. coli challenge conditions enhanced BW, BWG, and FCR. Differences in the amount of addition, duration of the experiment period, the selection of animals employed or the environmental circumstances could all be contributing factors to the variety in growth performance findings found in earlier investigations.

### Carcass traits

At 42 days of age, broiler chicks fed DPP-supplemented diets exhibited significantly lower live body weights compared to the control group (Table 3). Despite the reduced live weight, the relative weights of eviscerated and dressed carcasses were notably higher in the DPP-treated groups, suggesting a favorable redistribution of body mass due to the high phenol and flavonoid content in DPP as reported by (Kishawy et al. 2019). This shift likely reflects enhanced muscle deposition coupled with reduced fat accumulation, as indicated by the significantly lower abdominal fat percentages observed in DP*P-*supplemented birds. These results align with findings from Hamady et al. (2015), who reported increased dressed carcass percentages in broilers fed pomegranate peel extract. Similarly, Elbaz (2023) observed improved carcass dressing percentages with fermented pomegranate peel supplementation under heat-stress conditions, further supporting the potential of DPP to enhance carcass quality. Dietary DPP supplementation significantly influenced the relative weights of internal organs, with the effects most pronounced at the 2% inclusion level. Chicks in this group exhibited the highest relative weights for the liver, heart, gizzard, and spleen. Additionally, those receiving 1% DPP showed significantly increased gizzard and spleen weights compared to controls. These findings suggest that DPP supplementation promotes organ development, potentially due to the bioactive compounds and antioxidants present in pomegranate peels. Abbas et al. (2017) similarly reported increased liver and heart weights in quails when dietary corn was partially replaced with DPP at 2.5–7.5%. The antioxidant properties of phenols, flavonoids, and tannins in DPP may stimulate enhanced metabolic activity in these organs, leading to their increased relative sizes. A key benefit of DPP supplementation observed in this study was the significant reduction in abdominal fat percentage in the treated groups. This effect could stem from the bioactive compounds in DPP, which influence lipid metabolism. Ahmadipour et al. (2021) similarly reported that dietary inclusion of pomegranate at 7.5 and 10 g/kg significantly reduced abdominal fat deposition in broilers. This reduction in fat accumulation aligns with the hypothesis that pomegranate-derived compounds modulate energy balance and fat metabolism through their effects on hormones like leptin and adiponectin (Al-Muammar and Khan 2012).

**Table 3:** Effect of supplementing broiler diets with different levels of pomegranate peel on carcass characteristics at 42 days old

Variables	**C**	DPP1	DPP2	SEM	*P-*value
LBW	2636.17^a^	2363.33^b^	2370.83^b^	34.46	<.0001
Eviscerated carcass %	70.59^b^	71.68^ab^	72.35^a^	0.27	0.0194
Dressed carcass %	74.37^c^	75.66^b^	76.84^a^	0.32	0.0013
Liver%	2.10^b^	2.01^c^	2.23^a^	0.025	<.0001
Heart%	0.35^b^	0.37^b^	0.53^a^	0.020	<.0001
Gizzard%	1.33^c^	1.60^b^	1.73^a^	0.042	<.0001
Spleen%	0.12^c^	0.17^b^	0.14^a^	0.005	<.0001
Abdominal fat %	1.74^a^	1.14^b^	1.03^b^	0.079	<.0001

^a,^^b,c^ Different superscript letters within a row indicate significant differences between means at a specified significance level (*P* < 0.05 or *P* < 0.01). C: Control (basal diets); DPP1; basal diets supplemented with dried pomegranate peel at 1%.; DPP1; basal diets supplemented with dried pomegranate peel at 2%

### Blood parameters

The impact of dietary dried pomegranate peel (DPP) supplementation on blood biochemical parameters is shown in Table 4. A lipid profile is used to track and screen chronic cardiovascular disease in animals and the degree to which it affects avian production. In the current investigation, dietary DPP supplementation significantly reduced the levels of glucose, cholesterol, HDL, and triglycerides compared to the control group. Additionally, DPP supplementation slightly increased the total antioxidant capacity and reduced malondialdehyde levels, although these changes were not statistically significant compared to the control group. However, the current results indicate that incorporating DPP into the broiler diet had no significant effect on the levels of total protein, albumin, globulin, or liver enzymes.

**Table 4:** Effect of supplementing broiler diets with different levels of pomegranate peel on blood biochemical parameters

Parameters	C	DPP1	DPP2	SEM	*P-*value
Total protein	6.69	6.66	6.68	0.01	0.6963
Albumin	3.27	3.18	3.31	0.06	0.6924
Globulin	3.39	3.47	3.37	0.06	0.7628
Al/Gl ratio	0.97	0.93	0.99	0.04	0.7735
Glucose	233.76^a^	209.14^b^	214.53^b^	3.37	0.0018
Cholesterol	257.05^a^	144.82^b^	135.84^b^	14.69	<.0001
HDL	73.12^a^	51.25^b^	48.02^b^	3.08	<.0001
LDL	12.05^a^	9.08^b^	10.97^a^	0.41	0.0041
Triglyceride	176.15^a^	82.75^b^	80.05^b^	11.27	<.0001
AST	15.31	12.22	15.30	0.75	0.1495
ALT	209.20	182.17	204.07	6.34	0.1862
MAD	1.05	0.96	0.92	0.06	0.3612
TAOC	2.93	3.09	3.13	0.04	0.3572

^a,^^b,c^ Different superscript letters within a row indicate significant differences between means at a specified significance level (*P* < 0.05 or *P* < 0.01). C: Control (basal diets); DPP1; basal diets supplemented with dried pomegranate peel at 1%.; DPP1; basal diets supplemented with dried pomegranate peel at 2%

The lack of significant changes in total protein, albumin, globulin, and liver enzymes may indicate that DPP supplementation does not adversely affect liver function and protein metabolism in broilers. However, the observed decrease in serum cholesterol and triglyceride levels in the groups treated with pomegranate peel may be attributed to its potential to regulate lipid metabolism, as reported by Hou et al. (2019). This effect likely occurs through the inhibition of pancreatic lipase, as described by Lei et al. (2007), which suppresses lipid digestion and absorption. Consequently, these mechanisms could explain the reductions in body weight and weight gain (Table 2) and abdominal fat percentage (Table 3) observed in the treated groups. Our results align with those of Ahmadipour et al. (2021), who reported significant reductions in serum triglyceride and cholesterol concentrations when broiler diets included pomegranate peel at 7.5 and 10 g/kg. Similarly, Gosai et al. (2023) observed that dietary supplementation with DPP at levels of 0.25%, 0.5%, and 1% significantly reduced triglyceride and cholesterol concentrations compared to controls. However, they noted that DPP supplementation did not affect other parameters such as blood hemoglobin, hematocrit, red and white blood cell counts, glucose, total protein, albumin, HDL, LDL, SGPT, or SGOT. The slight increase in total antioxidant capacity and decrease in malondialdehyde (a marker of oxidative stress) are promising, indicating a potential protective effect of DPP against oxidative damage.

### Cecal microbiota

Effect of dietary dried pomegranate peel (DPP) supplementation on the cecal microbiota is shown in Table 5. Statistical analysis of the logarithm of the number of colony-forming units (CFU) revealed that DPP supplementation significantly reduced the counts of total bacteria, *E. coli*, and *Salmonella* compared to the control group. It is worth noting that no significant differences were observed among the treated groups in the counts of *E. coli* or *Salmonella*, while the total bacterial count in the group that received 2% DPP was significantly lower than in the group that received 1% DPP.

**Table 5:** Effect of supplementing broiler diets with different pomegranate peel on cecal microbiota

Variables	C	Dpp1	Dpp2	*P-*value
Total bacterial count	8.70 ± 0.25^a^	6.48 ± 0.18^b^	5.60 ± 0.11^c^	<.0001
*E. coli* count	5.80 ± 0.18^a^	3.42 ± 0.14^b^	4.00 ± 0.29^b^	<.0001
*Salmonella* count	5.56 ± 0.08^a^	3.75 ± 0.13^b^	3.53 ± 0.02^b^	<.0001

^a,^^b,c^ Different superscript letters within a row indicate significant differences between means at a specified significance level (*P* < 0.05 or *P* < 0.01). C: Control (basal diets); DPP1; basal diets supplemented with dried pomegranate peel at 1%.; DPP1; basal diets supplemented with dried pomegranate peel at 2%

The reduction in pathogenic bacteria observed in the groups treated with DPP can be attributed to the antimicrobial properties of the bioactive compounds abundantly present in DPP. Compounds such as phenolics, flavonoids, and tannins in pomegranate peel are well-documented for their ability to inhibit the growth of harmful microorganisms. Pomegranate peel contains a significant source of organic acids, including fumaric, citric, malic, acetic, oxalic, tartaric, lactic, and ascorbic acids, as well as numerous other nutrients (Poyrazoğlu et al. 2002). By acidifying the digestive tract and lowering the pH level in the surrounding environment, the organic acids may increase the chicken’s resistance to infections and make it more difficult for certain intestinal pathogens, such E. coli, to proliferate (Khan et al. 2022). According to studies, the amount of E. coli was considerably lower in the 4% pomegranate peel powder group than in the control group (*P* < 0.05) (Ghasemi-Sadabadi et al. 2021). Previous studies support these findings, demonstrating significant reductions in bacterial populations with the dietary inclusion of pomegranate byproducts. Hamady et al. (2015) reported a notable decrease in bacterial counts following pomegranate peel supplementation, while Xu et al. (2024) emphasized the role of pomegranate peel in maintaining a balanced intestinal microbiota, further promoting gut health.

### Histomorphometry of Bursa and Ileum

**Fig. 1:**
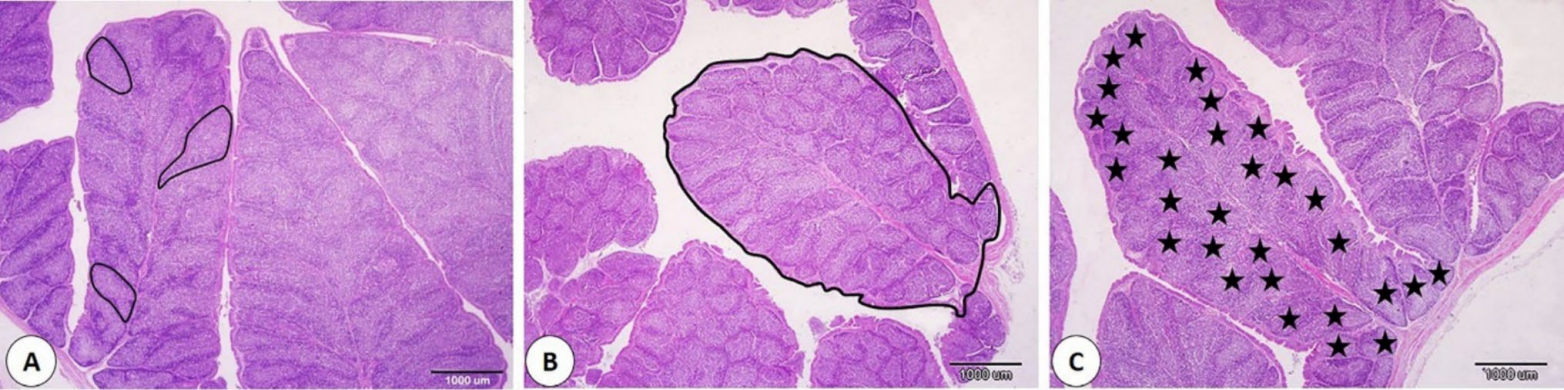
Effect of supplementing broiler diets with different pomegranate peel on histomorphometry of bursa of Fabricius. **A** Control group. **B** 1% O group. **C**) 2% O group. The bordered area in **A** showed follicle area, the bordered area in **B** showed area of plica, and the bordered area in **C** showed number of follicles

The bursa of Fabricius plays a critical role in B cell development and maintenance as it acts as a key primary lymphoid organ. B cells are essential for adaptive immunity (Ratcliffe 2006; Davani 2013; Çetin and Özaydın 2021). In the current study, histomorphometry investigated the impact of dietary DPP supplementation on the bursa. Histological analysis revealed that the 2% DPP group exhibited a significant increase in the plica area compared to the control and 1% DPP groups, suggesting that higher DPP levels may promote bursal tissue growth (Table 6 and Fig. 1). In contrast, the 1% DPP group showed a higher follicle number per plica than the control and 2% DPP groups, indicating that lower levels of DPP enhance follicular proliferation. Despite the differences in the area of plicae and follicular number, no significant changes were detected among the groups in the follicular area. This recommends that DPP supplementation may not affect the size directly of individual follicles but influences the number of follicles within the plica. These results emphasize the complex association between DPP and follicular dynamics in the bursa. Our data revealed that the use of DPP enhances the immune system of birds through the improvement of the histomorphometrical character of the bursa. Several studies evaluated the effect of DPP in diet and considered it a dietary constituent to improve the immune system (Saeed et al. 2018; Hosseini-Vashan and Raei-Moghadam 2019). DPP can regulate the cytokines, transcription factor production, and act as antioxidants that enhance the immune system by facing the free radicals produced throughout the immune responses (Lipiński et al. 2017).

**Table 6:** Effect of supplementing broiler diets with different pomegranate peel on histomorphometry of bursa and ileum

Variables	C	DPP1	DPP2	SEM	*P-*value
plica Area/um	12538332^b^	11461927^b^	16008214^a^	717,965	0.0007
No. of follicles/plica	27.00^b^	33.67^a^	25.67^b^	1.36	0.0053
Follicle area/um	604678^a^	526519^a^	558502^a^	16,297	0.1370
Villi length (um)	814.77^ab^	687.53^b^	956.16^a^	44.746	0.0153
Crypt length (um)	225.03^b^	382.25^a^	251.31^b^	25.26	0.0004
Muscular layer thickness (um)	244.13^ab^	263.67^a^	240.90^b^	4.68	0.0757
Epithelium thickness (um)	44.77^a^	39.43^a^	44.86^a^	1.44	0.2309

^a,^^b^ Different superscript letters within a row indicate significant differences between means at a specified significance level (*P* < 0.05 or *P* < 0.01). C: Control (basal diets); DPP1; basal diets supplemented with dried pomegranate peel at 1%.; DPP1; basal diets supplemented with dried pomegranate peel at 2%

**Fig. 2:**
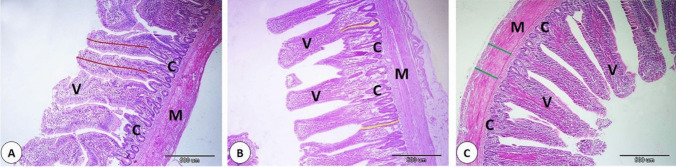
Effect of supplementing broiler diets with different pomegranate peel on histomorphometry of ilium. **A** Control group. **B** 1% P group. **C**) 2% P group. The red line in **A** showed length of the villi, the yellow line in **B** showed depth of the crypt, and the green line in **C** showed thickness of the muscle layer

Our data clarify the impact of dietary DPP supplementation on ilium histomorphometry, revealing distinct changes in several ilium parameters (Table 6 and Fig. 2). The findings suggest that DPP intake plays an important role in regulating intestinal structure, potentially influencing gut function. The 2% DPP group revealed significantly longer villi compared to the control and 1% DPP groups, representing that higher DPP levels may encourage villus growth. This aligns with the villi’s role in the absorption of nutrients, as longer villi offer a greater surface area for absorption. The increased villus length in the 2% DPP group, suggests that DPP supplementation can enhance villous elongation through enterocyte proliferation. On the other hand, the 1% DPP group showed a significant increase in the depth of the intestinal crypts compared to the control and 2% DPP groups, suggesting that lower DPP levels may induce crypt cell proliferation. The muscular layer thickness was greater in the 1% DPP group, indicating that lower DPP intake might enhance the development of the muscular layer and potentially affect intestinal motility and digestion. On the other hand, the epithelial lining of the villi showed no significant differences among different groups, indicating that DPP supplementation did not directly affect epithelial thickness under the experimental conditions. Our data agrees with previous studies on the enhancing effect of dietary supplementation of DPP on gut function through improving the histomorphometrical parameters such as villi length and crypt depth (Mahmud et al. 2016; Abbas et al. 2017; Maqsood et al. 2024).

## Conclusion

The present study demonstrated that dietary supplementation with dried pomegranate peel (DPP) influenced broiler performance, carcass traits, blood parameters, cecal microbiota, and histomorphometry of the bursa and ileum. While DPP inclusion led to a reduction in body weight and weight gain, it improved feed efficiency, particularly at the 2% supplementation level. Additionally, broilers fed DPP exhibited improved carcass composition, with higher relative carcass weights and lower abdominal fat percentages. Blood biochemical analysis revealed that DPP supplementation significantly reduced glucose, cholesterol, HDL, and triglyceride levels, with slight improvements in antioxidant capacity. The cecal microbiota analysis highlighted the antimicrobial potential of DPP, as it significantly reduced total bacterial, E. coli, and Salmonella counts, particularly at the 2% inclusion level. Histomorphometric findings indicated that DPP positively influenced immune and intestinal structures, enhancing bursal development, villus length, and crypt depth in a dose-dependent manner.

Overall, these findings suggest that DPP can serve as a functional feed additive with potential benefits for gut health, immune response, and carcass traits. However, its impact on growth performance warrants further investigation to optimize dietary inclusion levels for achieving both health benefits and optimal productivity in broiler production.

## Data Availability

The datasets generated and/or analyzed during the current study are available from the corresponding author on reasonable request.
